# Validating Visual Stimuli of Nature Images and Identifying the Representative Characteristics

**DOI:** 10.3389/fpsyg.2021.685815

**Published:** 2021-09-10

**Authors:** Terri Menser, Juha Baek, Jacob Siahaan, Jacob M. Kolman, Domenica Delgado, Bita Kash

**Affiliations:** ^1^Center for Outcomes Research, Houston Methodist, Houston, TX, United States; ^2^Department of Population Health Sciences, Weill Cornell Medical College, New York, NY, United States; ^3^Center for Innovation, MD Anderson Cancer Center, Houston, TX, United States; ^4^Department of Health Policy and Management, Texas A&M University, College Station, TX, United States

**Keywords:** nature therapy, ecotherapy, functional magnetic resonance imaging, image validation, validation study

## Abstract

This study fills a void in the literature by both validating images of nature for use in future research experiments and examining which characteristics of these visual stimuli are found to be most representative of nature. We utilized a convenience sample of university students to assess 129 different nature images on which best represented nature. Participants (*n* = 40) viewed one image per question (*n* = 129) and were asked to rate images using a 5-point Likert scale, with the anchors “best represents nature” (5) and “least represents nature” (1). Average ratings across participants were calculated for each image. Canopies, mountains, bodies of water, and unnatural elements were identified as semantic categories of interest, as well as atmospheric perspectives and close-range views. We conducted the ordinary least squares (OLS) regression and the ordered logistic regression analyses to identify semantic categories highly representative of nature, controlling for the presence/absence of other semantic categories. The results showed that canopies, bodies of water, and mountains were found to be highly representative of nature, whereas unnatural elements and close-range views were inversely related. Understanding semantic categories most representative of nature is useful in developing nature-centered interventions in behavioral performance research and other neuroimaging modalities. All images are housed in an online repository and we welcome the use of the final 10 highly representative nature images by other researchers, which will hopefully prompt and expedite future examinations of nature across multiple research formats.

## Introduction

Natural settings have been shown to improve various health outcomes (Berman et al., 2014; Berto, 2014; Franco et al., 2017) and can be important to one’s overall well-being (Capaldi et al., 2015; Dean et al., 2018). Studies have examined nature’s effect on recovery in a variety of patient populations including cancer (Blaschke, 2017), dementia (Uwajeh et al., 2019), and surgery (Ulrich, 1984; Ulrich et al., 1993; Diette et al., 2003). The health outcomes from those studies found associations between exposure to nature and the ability to cope with illness (Blaschke, 2017), decreased stress and anxiety (Bratman et al., 2019), improved cognitive functioning (Uwajeh et al., 2019), and decreased pain (Ulrich, 1984; Ulrich et al., 1993; Diette et al., 2003). Furthermore, exposure to commonly accessible natural settings such as forests (Hansen et al., 2017) and blue spaces (i.e., areas in close proximity to bodies of water) (Gascon et al., 2017) have been shown to have a positive association with health. Forest therapy has been shown to improve well-being (Hansen et al., 2017), mental health in psychiatric patients (Bielinis et al., 2019), sleep quality in cancer patients (Kim et al., 2019), and physiological improvements in patients coping with chronic widespread pain (Han et al., 2016).

Common physiological dependent variables that assess nature’s effects are shifts in blood pressure, heart rate, and cortisol levels in order to evaluate decreases in stress and possible reduction of cardiovascular disease risk (Haluza et al., 2014; Twohig-Bennett and Jones, 2018; Mygind et al., 2019). Psychological factors that are often studied in research focused on nature’s effects include self-assessments of mental health components (e.g., changes in concentration, stress levels, etc.) (Bowler et al., 2010; Haluza et al., 2014). Both physiological and psychological responses can give insight to the quantification of effective nature exposure doses (Barton and Pretty, 2010; Cox et al., 2017; Browning et al., 2020). However, the management of experimental conditions in natural settings are a common obstacle when assessing nature’s effects in outdoor settings of the true environment (Tennessen and Cimprich, 1995; Bowler et al., 2010). As a result, researchers have utilized artificial, indoor settings (Jo et al., 2019), and visual stimuli such as images (Gamble et al., 2014; Van den Berg et al., 2016), videos (Pilotti et al., 2014; McAllister et al., 2017; Bourrier et al., 2018), and virtual reality (Valtchanov et al., 2010; Calogiuri et al., 2018; Browning et al., 2020) for controlled nature experiments.

An initial challenge to conducting such studies is definitional. “Nature” is a metaphysically troubling concept, complicated by debates on socially constructed meaning and broad conceptions which deconstruct the distinction between the “natural” and “human” (Frumkin et al., 2017). The current Medical Subject Headings (MeSH) term “Nature” is maximally broad, “restrict[ed] to Nature as an abstract or philosophical concept” as “the system of all phenomena in space and time; the totality of physical reality” (U.S. National Library of Medicine, 2020), and is not indexed with terms related to the environment or ecology. Research agendas on nature and human health may eschew such philosophical quandaries in favor of practical, example-based definitions, including elements such as “plants and non-human animals … together with abiotic elements such as sunset or mountain views” and settings ranging from managed parks to so-called “wilderness” (Frumkin et al., 2017, p. 1). Indeed, example-based selections of stimuli do “reflect practical experimental needs” over theory (Zhang et al., 2019, p. 1180). However, given the potentially wide range of disparate intuitions about what is natural, and in particular the complex challenges of producing rigorous and reproducible functional magnetic resonance imaging (fMRI) studies (Poldrack et al., 2008), the construct validity of pictorial stimuli purportedly representing nature should be tested. For instance, Berman et al. (2014) used survey methods in which participants rated nature scenes for similarity, provided unprompted single-term labeling (including nature/natural, manmade, etc.) to images, and rated images numerically for degree of naturalness, with an analysis to correlate the high-naturalness scenes with common low-level visual features (saturation, hue, brightness, entropy, gradient, and straight vs. curved edge density) that may be indicative of such scenes in general.

The use of fMRI technology with validated nature images offers a means of examining nature’s effect on the brain in a controlled setting with replicable results. Few studies have taken advantage of fMRI methodology to better understand neurological responses to nature (Kim et al., 2010a,b; Bratman et al., 2015); fMRI studies have largely focused on patterns in scene recognition and discrimination between subtypes of semantic categories of scenes. Walther et al. (2009) identified regions of the brain associated with distinguishing types of natural environment (beaches, buildings, forests, highways, industry, and mountains). Park et al. (2011) and Kravitz et al. (2011) likewise found a family of visual areas involved in scene recognition and spatial factors associated with scene recognition, in part using images depicting natural scenery.

There are limited studies that offer validated nature images for experimental use. The SYNS dataset (SYNS dataset, 2015; Adams et al., 2016) uses a variety of nature-related subcategories informed by land use categories in the United Kingdom, but the underlying study’s focus on low-level feature analysis (surface attitude), specifically for image-3D perceptual relations, did not seem to involve or require an independent validation step of global semantic categorization. There are also a variety of databases containing validated image stimuli, but not necessarily indexed by or validated for “naturalness” semantic categories, including: food behaviors (King et al., 2018), morals (Crone et al., 2018), fears (Michałowski et al., 2017), and disgust (Haberkamp et al., 2017). The purpose of this study is to validate images of nature, as a semantic category, and secondarily to identify semantic subcategories found to be most representative. The availability of these images both expedites and encourages future examinations of neuroactivity in response to nature exposure, and can facilitate a range of other research designs focused on nature.

## Materials and Methods

### Study Participants and Image Selection

Images of nature were selected by two researchers (TM and DD) from four open source websites using six search terms (refer to Supplementary Appendix 1 for complete details) in addition to searching the International Affective Picture System (Lang et al., 1997) for both nature and urban images; the latter contributed only to the control set of urban visual stimuli. The resulting 129 nature images were presented as an online survey, open for 30 days from January 21 to February 20, 2019, to a total of 40 undergraduate students, medical students, and public health graduate students from the Texas A&M University system. Table 1 shows the composition of students from our recruitment pool within the Colleges of Engineering, Medicine, and Public Health from fall of 2018 (Texas A&M University, 2020). We provided compensation to the participants for their time in the form of a $20 gift card. The study protocol was approved by the Houston Methodist Research Institute’s Institutional Review Board (Pro00020819).

**TABLE 1:** Demographics of the Texas A&M University Student Population for Colleges of Engineering, Medicine, and Public Health (2018).

	**Gender**		**Ethnicity**				
**Background**	**Male**	**Female**	**White**	**Hispanic**	**Asian**	**Black**	**Other**
Population (%)	16,597 (74)	5,829 (26)	10,393 (46.34)	4,911 (21.90)	3,046 (13.58)	656 (2.93)	3,420 (15.25)

### Procedures

Participants viewed the survey which consisted of the selected images and rated each one based on a 5-point Likert scale, with “1” corresponding to “least representing nature” and “5” to “best representing nature.” A researcher (JS) reviewed each image to make a list of the major features in the image. Then, two researchers (TM and JS) condensed these images into categories and features that aligned with previous restorative health literature, noting the established divide between blue and green space. While this further subcategorization was subjective, the coders had no pre-established concept of what elements would be predictive of high ratings of naturalness which is what prompted the *post hoc* analysis. JS initially coded the images, guided by TM; to confirm the categorizations, another researcher (JB) reassessed the 129 images any differences in coding were decided by TM.

**FIGURE 1:**
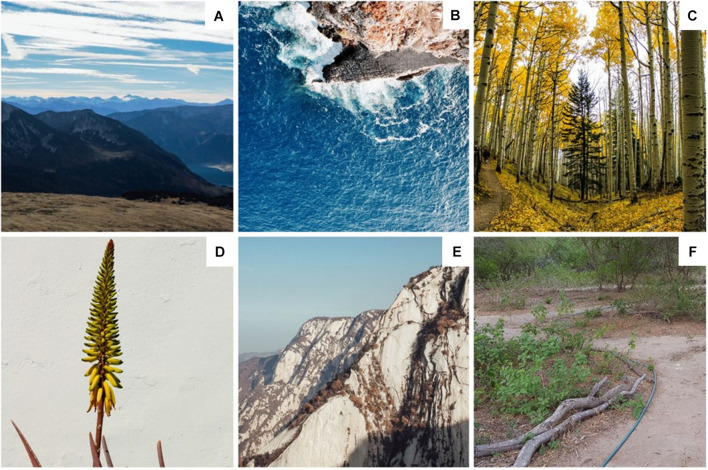
Semantic and framing features. **(A)** Atmospheric perspectives, **(B)** bodies of water, **(C)** canopies, **(D)** close-range views, **(E)** mountains, and **(F)** unnatural elements.

As a result, the research team identified semantic subcategories – bodies of water, canopies (vegetation over eight feet tall), mountains, unnatural elements (i.e., objects and man-made structures, such as boats and walkways, respectively) – and image framing properties – atmospheric perspectives and close-range views – that were coded for each image to conduct *post hoc* analyses to discern which features were predictive of receiving a high rating for nature representation as shown in Figure 1. An atmospheric perspective was defined as scenery where objects are perceived as distant when the scattering of lights blurs the outline of objects which could make distant mountains appear blue and more nearby mountains appear clear (Kalloniatis and Luu, 1995). Close-range views were considered a view focused on a singular object or small area (e.g., flowers, plants, etc.). We formed three levels of representations based on naturalness Likert rating: high (top 25%), moderate (25–75%), and low (75% and below). The top 10 highly representative nature images used for our fMRI study were selected based on the naturalness ratings displaying the semantic categories and image properties identified as representative of nature.

### Data Analysis

Descriptive statistics were used to calculate mean, minimum, and maximum values of naturalness scores by semantic category. A two-sample *t*-test was performed to compare average naturalness scores between images that include a semantic category and those that did not include that semantic category. We conducted the multivariate ordinary least squares (OLS) regression and multivariate ordered logistic regression (OLR) analyses to identify semantic categories highly representative of nature, controlling for the presence/absence of other categories. In the OLS model, the dependent variable was a continuous variable of naturalness score and the independent variables were all semantic categories coded in a binary manner (presence of a semantic category: 1 or absence: 0). The results were represented as coefficients and 95% confidence intervals (CIs). In addition, the OLR model had a dependent variable with three categories (high, moderate, and low) of naturalness scores and all independent variables included in the OLS model. The odds ratios (ORs) and 95% CIs were presented in this model. Statistical analyses were conducted using Stata version 15 (StataCorp LLC, College Station, TX, United States). All statistical tests were two-sided, and a *P*-value < 0.05 was considered statistically significant.

## Results

The average value of ratings for 129 nature images was 4.14 (standard deviation = 0.49, minimum value = 2.73, maximum value = 4.88). Table 2 shows the descriptive statistics of the number, average score of naturalness ratings, and levels of naturalness (high, moderate, and low) by semantic subcategory or framing property. We found that canopies (*N* = 76) and bodies of water (*N* = 66) were the most common throughout all images while atmospherics perspectives (*N* = 25) and close-range views (*N* = 25) were the least common. Unnatural features (mean = 3.76) and close-range views (mean = 3.66) scored the lowest naturalness ratings, whereas mountains (mean = 4.49), atmospheric perspectives (mean = 4.41), bodies of water (mean = 4.37), and canopies (mean = 4.27) had higher scores than the average naturalness ratings (4.14).

**TABLE 2:** Average scores of naturalness ratings and levels of naturalness by semantic subcategory or image property.

	***N***	**Average score of naturalness**	**Level of naturalness (*N*, %)**		
			**High (top 25%)**	**Moderate (25–75%)**	**Low (≤75%)**
**Semantic subcategories**					
Canopies	76	4.27	28 (36.8)	35 (46.1	13 (17.1)
Mountains	43	4.49	25 (58.2)	17 (39.5)	1 (2.3)
Bodies of water	66	4.37	26 (39.4)	36 (54.5)	4 (6.1)
Unnatural elements	28	3.76	0 (0.0)	15 (53.6)	13 (46.4)
**Image properties**					
Atmospheric perspectives	25	4.41	13 (52.0)	10 (40.0)	2 (8.0)
Close-range views	25	3.66	0 (0.0)	7 (28.0)	18 (72.0)
Total	129	4.14	34 (26.4)	62 (48.1)	33 (25.6)

The two-sample *t*-tests showed that all semantic categories and framing properties had a significant difference in naturalness ratings when compared to images without them (Table 3). Particularly, nature images that included canopies, mountains, bodies of water, and atmospheric perspectives had significantly higher average scores than those that did not include them, respectively (*P* < 0.001). On the other hand, average scores of naturalness ratings were significantly lower in nature images with unnatural elements and close-range views than those without these elements (*P* < 0.001).

**TABLE 3:** Comparison of average naturalness scores between presence and absence by semantic subcategory or image property.

	**Presence**		**Absence**		***P*-value**
	***N***	**mean (min, max)**	***N***	**mean (min, max)**	
**Semantic subcategories**					
Canopies	76	4.27 (2.73, 4.88)	53	3.95 (2.82, 4.87)	<0.001
Mountains	43	4.49 (3.73, 4.88)	86	3.96 (2.73, 4.87)	<0.001
Bodies of water	66	4.37 (2.89, 4.88)	63	3.90 (2.73, 4.69)	<0.001
Unnatural elements	28	3.76 (2.73, 4.43)	101	4.24 (3.34, 4.88)	<0.001
**Image properties**					
Atmospheric perspectives	25	4.41 (2.89, 4.76)	104	4.08 (2.73, 4.88)	<0.001
Close-range views	25	3.66 (2.89, 4.44)	104	4.25 (2.73, 4.88)	<0.001

Table 4 shows the results of multivariate OLS regression and multivariate OLR analyses. We found that canopies, mountains, and bodies of water were positively associated with naturalness ratings in both models, consistently, indicating that these semantic categories are highly representative of nature. In addition, unnatural elements and close-range views were found to be negatively associated with ratings of nature representation. However, atmospheric perspectives were not significant in these models.

**TABLE 4:** Results of multivariate ordinary least squares (OLS) regression and multivariate ordered logistic regression (OLR) models.

**Semantic subcategory/image property**	**Multivariate OLS model**		**Multivariate OLR model**	
	**Coef. (95% CI)**	***P*-value**	**OR (95% CI)**	***P*-value**
Canopies	0.25(0.12,0.38)	<0.001	4.38(1.43,13.4)	0.010
Mountains	0.32(0.18,0.47)	<0.001	13.99(3.8,51.49)	<0.001
Bodies of water	0.29(0.18,0.41)	<0.001	6.97(2.55,19.09)	<0.001
Unnatural elements	−0.61(−0.74,−0.48)	<0.001	0.02(0.01,0.07)	<0.001
Atmospheric perspectives	−0.03(−0.20,0.14)	0.726	0.83(0.19,3.51)	0.798
Close-range views	−0.26(−0.44,−0.08)	0.005	0.09(0.02,0.52)	0.007

*N = 129; Coef, coefficient; OR, odd ratio; 95% CI, 95% confidence interval.*

Finally, the principal investigator (TM) selected the final 10 images among the highest scoring 31 images scored 4.6 or higher from the total of 129 images rated to be used in a related project. All of these images and their respective ratings are available for future research and are housed in an online repository (Supplementary Appendix 2; see data availability statement). Table 5 describes the average naturalness ratings, and semantic categories included in the set of 10 highly representative nature images. The average natural ratings of the image set ranged between 4.62 and 4.88 with 6 out of 10 images including all three semantic categories: canopies, mountains, and bodies of water.

**TABLE 5:** Top 10 highly representative nature images, average naturalness ratings, and semantic categories.

**Rank**	**Image number**	**Average naturalness**	**Canopies**	**Mountains**	**Bodies of water**
1	108	4.88	✓	✓	✓
2	7	4.87	✓	✓	✓
3	37	4.87	✓	✓	✓
4	87	4.76	✓	✓	✓
5	88	4.75	✓	✓	✓
6	112	4.72	✓	✓	✓
7	34	4.70			✓
8	56	4.69	✓	✓	
9	55	4.63		✓	✓
10	17	4.62			✓

## Discussion

This study yielded a set of validated nature images to be used in nature-based research including fMRI methodological approaches. These images are available to researchers^1^ to encourage the study of nature’s effects and to minimize the initial outlay of resources and decrease the time required to conduct nature studies. The semantic subcategories that were found to be most predictive of high ratings of nature representation included canopies, bodies of water, and mountains which aligns with the limited studies available in the current literature on nature in these areas.

High naturalness scores for open spaces and atmospheric perspectives, and low naturalness for close-range views, align with studies that have considered the openness vs. closedness of spatial boundaries as important mediators to consider, both in survey-based scene classification (Zhang et al., 2019), and in fMRI studies of brain activity (Kravitz et al., 2011; Park et al., 2011). Zhang et al. (2019) in particular found a high correlation between natural-open and between manmade-closed; relative distance (i.e., nearness/farness of the exemplar terrain in how the shot is framed) is also a factor to consider (Kravitz et al., 2011). Other lower-level image properties should also be considered in image selection; for instance, correlations have been found between images rated as highly natural and edge density, fewer straight and more curved edges, and “less hue diversity” (Berman et al., 2014, p. 17). Note that unlike much of this literature, we remained agnostic regarding whether or not these nature images constitute “scenes” as such, but there are valuable related discussions of the role of natural scene recognition *qua* scene in the literature, and related low-, mid-, and high-level visual features which contribute to the perception of scenes (Groen et al., 2017).

The high representation of canopies coheres with the restorative effects that have been discussed through specifically forest therapy as a type of nature exposure. Forest therapy, defined as having interaction in the forest (e.g., walks, exercises, etc., in the forest environment) has also been shown to result in positive changes in well-being (Shin et al., 2010) and has resulted in a feeling of restoration when compared to an urban environment (Takayama et al., 2019). There are also multiple theories that canopies (i.e., vegetation over eight feet tall) are a feature that can support a viewer’s ability to learn about an environment (Hunter and Askarinejad, 2015). Canopies can provide a sense of organizational symmetry with the pairing of trees that could encourage a viewer to explore the environment even further (Hunter and Askarinejad, 2015).

The association of bodies of water with high levels of nature representation is likely related to its restorativeness; a prior study found a higher likelihood of participants rating both natural and man-made scenery to have higher perceived restorativeness when water elements were present (White et al., 2010). Additionally, there has been fairly extensive studies to understand the benefits of blue space (i.e., areas near bodies of water) – a systematic review of 35 quantitative studies on blue spaces found consistent positive association with well-being and mental health (Gascon et al., 2017).

The inclusion of mountains in the higher-rated images can be explained by the awe-evoking effects that have been reported in the literature. Although there are no known therapeutic applications of the mountain sceneries, studies have examined participants’ differential responses to mountain scenery and neutral nature settings, finding that mountain scenery conveys a higher degree of vastness (Joye and Bolderdijk, 2015). The lack of studies focused on the effects of exposure specifically to time in the mountains is likely due to the inaccessibility of this environment, but our results show that this feature of nature is highly predictive of ratings of nature representation.

We encourage the use of these validated images to promote the use of fMRI technology to discern neurological responses to better understand the effects of nature. For nature studies, controlling for conditions to assess and accurately quantify the results can be difficult to do in the natural environment (Velarde et al., 2007); fMRI studies offer an opportunity to alleviate the difficulties in the quantification of subjective results. Lack of available validated images from fMRI studies encumbers reproducible science (Eklund et al., 2016; Munafò et al., 2017; Poldrack et al., 2017) which limits scientific progress in understanding the benefits of nature. Additionally, the availability of validated images can help to identify factors that cause false-positive fMRI results (Bennett et al., 2009; Eklund et al., 2012). The use of fMRIs and images of natural and urban sceneries have identified neurological responses that suggested that human beings have an inherent preference for living in natural environments (Kim et al., 2010a,b; Bratman et al., 2015). In addition, researchers found that viewing water sceneries after urban sceneries enabled activation systems (Tang et al., 2017). Understanding the restorative effects of natural environments, and more abstractly of visual features associated with natural environments (Berman et al., 2014), is useful in developing nature-centered interventions, and for utilizing fMRI technology for examinations of neuroactivity. Future work in using these images should also account for regions normally activated by natural scene recognition as identified in prior literature (Walther et al., 2009; Kravitz et al., 2011; Park et al., 2011), which can both aid in interpretation of results and also corroborate or challenge the survey-based ratings of these scenes as exemplifying the semantic category of “natural” and associated subcategories.

A limitation of this study is how our questionnaire only assessed nature representation for the nature stimuli and did not use the same scale for the urban images that were made available as a control; instead we assessed how representative the control images were of an urban environment which has previously been used as the visual stimuli for the control group in nature studies (Kim et al., 2010a,b). A second limitation is how our study may not be representative for a general population as recruitment was university-based (partially mitigated by the diversity of the particular university used). Choosing this recruitment method was a tradeoff which allowed for transparent reporting of overall population demographics of the recruitment pool even when individual demographics could not be obtained; this is in contrast to methods utilizing Amazon Mechanical Turk (Xiao et al., 2016; Zhang et al., 2019), which can affordably recruit a larger participant pool for image rating tasks, but with variable population demographics and with the added need to filter out frequent cases of “bad actors” either through intermittent trial surveys or by a response-outlier cutoff. Third, our study sought to validate images for specific experimental purposes in restorative health studies; others (Zhang et al., 2019) have noted that the use of a limited number of example subtypes of natural scenes can limit more theoretical explorations of global properties, and this limitation should be kept in mind when applying the image set presented here. Fourth, we have not filtered, normalized, or evaluated the role of low-level visual features in our image set, as others (Berman et al., 2014; cf. Groen et al., 2017) have done, which would be of interest particularly in the design of artificial scenes or built environments to mimic the qualities associated with images rated highly for naturalness. We left open that our survey responses may align with or deviate from these expected correlations.

Further studies should utilize stimuli that have been validated for nature representation in order to assess nature’s restorative effects and its mechanisms. More studies should also identify neurological responses in addition to shifts in emotional states such as awe, stress relief, and well-being in patient populations, as distinct from neutral scene-recognition neurological responses. Understanding changes in neuroactivity and emotions could aid in the implementation of therapeutic interventions that can tailor to the specific needs of patients.

## Data Availability Statement

The datasets presented in this study can be found in online repositories. The names of the repository/repositories and accession number(s) can be found below: https://osf.io/spf8y/?view_only=ea16da9ea27c486eb9f8997fd67ee897.

## Ethics Statement

The studies involving human participants were reviewed and approved by the Houston Methodist Research Institute’s Institutional Review Board. Written informed consent for participation was not required for this study in accordance with the national legislation and the institutional requirements.

## Author Contributions

TM and BK were responsible for the conception of this the study. TM and DD contributed to the study design and survey content. JK formed the participant survey and tracked participation. JB and JS conducted the statistical analyses for this study under TM’s guidance and supervision. TM, JB, and JS drafted the manuscript. JK, DD, and BK contributed to manuscript drafts and edits. TM, JB, and JK were responsible for manuscript revision. All authors read and approved the submitted version.

## Conflict of Interest

The authors declare that the research was conducted in the absence of any commercial or financial relationships that could be construed as a potential conflict of interest.

### Publisher’s Note

All claims expressed in this article are solely those of the authors and do not necessarily represent those of their affiliated organizations, or those of the publisher, the editors and the reviewers. Any product that may be evaluated in this article, or claim that may be made by its manufacturer, is not guaranteed or endorsed by the publisher.

## Footnotes

1
https://centerforhealthandnature.org/research/fmri-image-validation/
