# Supplementary figures and images for: Validating Visual Stimuli of Nature Images and Identifying the Representative Characteristics
Source: Front Psychol. 2021 Sep 10;12:685815. doi: 10.3389/fpsyg.2021.685815 (PMC8460908; doi:10.3389/fpsyg.2021.685815)

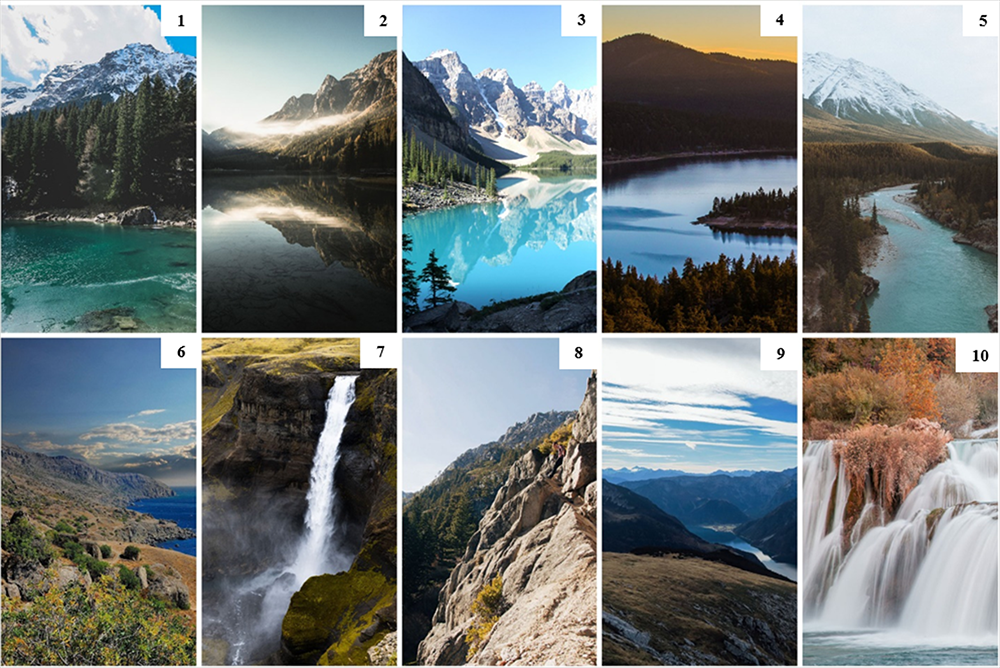

Supplement: Supplementary file 1 [file Image_1.TIF]
