# Supplementary material for: Validating Visual Stimuli of Nature Images and Identifying the Representative Characteristics
Source: Front Psychol. 2021 Sep 10;12:685815. doi: 10.3389/fpsyg.2021.685815 (PMC8460908; doi:10.3389/fpsyg.2021.685815)
Supplement: Supplementary file 2 [file Table_1.PDF]

Websites Used	***Nature Image Keywords***
**www.pixabay.com**	nature
**www.lifeofpix.com**	garden
**www.unplash.com**	hill
**www.canva.com**	forest
	park
	beach
